# Assessing Gender Differences in Caregiver Strain and Activities Among Older Adult Spousal Caregivers

**DOI:** 10.1093/geroni/igaf122.924

**Published:** 2025-12-31

**Authors:** Gilbert Gimm, Yiqing Qian, Mary Louise Pomeroy, Katherine Ornstein

**Affiliations:** George Mason University, Fairfax, Virginia, United States; Johns Hopkins University School of Nursing, Baltimore, Maryland, United States; Johns Hopkins Bloomberg School of Public Health, Baltimore, Maryland, United States; Johns Hopkins University, Baltimore, Maryland, United States

## Abstract

Male spousal caregivers have received less attention in the aging literature despite playing an important and growing role. Although prior studies have found male adult caregivers have lower average burden scores compared to female adult caregivers, less is known about whether such gender differences are associated with caregiving activities or hours. This study examined differences in caregiving activities and hours of male vs. female spousal caregivers of community-dwelling Medicare beneficiaries. Using nationally representative data on 451 spousal caregivers from Round 12 of the National Study of Caregiving (NSOC), we conducted weighted bivariate analyses to assess differences in caregiving roles by gender. We found that males represented almost one-half (45.5%) of the 5.2 million spousal caregivers. Among racial/ethnic subgroups, only non-White Hispanics had a larger share of male vs female spousal caregivers (12.8% vs. 6.3%). Although male spouses were less likely to experience physical strain (18.6% vs. 60.4%, p < 0.01) and emotional strain (18.7% vs 37.9%, p < 0.01) than female spouses, average caregiving hours did not significantly differ by gender. For caregiving activities, male spouses were more likely to provide transportation assistance than female spouses (81.9% vs. 61.4%, p < 0.01), but were less likely to assist with health behavior management (52.3% vs 69.8%, p < 0.05) or health system logistics (46.9% vs 79.6%, p < 0.01). Overall, males represented nearly one-half of spousal caregivers. These results suggest the need for additional studies on male caregiver activities and non-pharmacological interventions (online peer support) that can be tailored to address the unique experiences and needs of male caregivers.

